# Synthesis of the modified SBA-15 mesoporous silica with TAN ligand to preconcentrate and determine trace amounts of Ni (II) ions in water and wastewater samples

**DOI:** 10.3389/fchem.2024.1410136

**Published:** 2024-09-30

**Authors:** Taha Amouzad Mahdirajeh, Ali Mirabi, Mohammad Habibi Juybari, Ramin Zafar Mehrabian, Hamid Reza Jalilian

**Affiliations:** ^1^ Department of Chemistry, Islamic Azad University, Gorgan, Iran; ^2^ Department of Chemistry, Islamic Azad University, Qaemshahr, Iran

**Keywords:** nickel preconcentration, 1-(2-thiazolylazo)-2-naphthol/SBA-15, modification, mesoporous, silica

## Abstract

The synthesis of modified mesoporous silica (SBA-15), a powerful and highly effective adsorbent, was provided for the preconcentration, extraction, separation, and determination of trace amounts of Ni (II) ions. In this method, SBA-15 modified with 1-(2-thiazolylazo)-2-naphthol (TAN) was used as a suitable absorbent. The absorption of Ni (II) ions was studied using the FAAS technique. Transmission electron microscopy (TEM), thermogravimetric analysis (TGA), Brunauer–Emmett–Teller (BET), energy-dispersive spectroscopy (EDS), Fourier-transform infrared (FT-IR) spectroscopy, and CHNS elemental analysis techniques were used to assess the characteristics of SBA-15 and TAN/SBA-15. Effective parameters (pH, amount of nanocomposite, extraction time, and type of recovery solvent) for extracting Ni (II) ions via TAN/SBA-15 were investigated. The merit figures of the method were obtained with appropriate results, such as detection limit, accuracy, enrichment factor, and preconcentration factor. The calibration curve was linear within the range of 5.0–50 ng mL^-1^ with a detection limit of 1.8 ng mL^-1^. High preconcentration, small relative standard deviations, and enrichment factors (approximately 100) were represented by the statistical analysis. The proposed method for measuring Ni (II) ions was used at trace levels in real samples, such as seawater, river water, well water, and wastewater from a textile factory, an electrical power station, an MDF factory, and a food industrial company, with satisfactory results.

## Introduction

Nowadays, the pollution of natural waters by heavy metals has created a high level of concern owing to their potentially toxic impacts on living organisms. Heavy traffic, industrial development, and urbanization contaminate waters via heavy metals (Luiz Silva et al., 2009; Ghaedi et al., 2010; Lertlapwasin et al., 2010). Ni is a fairly toxic element compared to other metals. Nickel as a water pollutant has attracted a huge deal of attention because of its toxicity in low concentrations. It is an essential component of some enzymes. Tea is another fairly rich source of Ni, with 7.6 mg kg^−1^ of dried leaves (Templeton, 1996; Nielsen et al., 1999). The trace quantity of nickel is toxic or essential based on its concentration range. For example, nickel is apparently essential for appropriate liver function based on studies on rats and chicks (genetically, the former of which are relatively close to humans) (Manzoori and Bavali-Tabrizi, 2003; Safavi et al., 2004; Safavi et al., 2006). Nickel was essential to some domestic animals and plants. However, until 1975, it was not considered a biologically important metal, when Zerner found that urease is an enzyme containing nickel (Zerner, 1991; Thauer, 2001). Inhalation of Ni and its compounds is known to result in serious problems, such as nasopharynx, respiratory system cancer, malignant tumors, and lung and dermatological diseases (Templeton, 1996). Furthermore, Ni can result in a skin disorder known as an allergic reaction and nickel eczema (Kristiansen et al., 2000), and definite Ni compounds may be carcinogenic (Rezaee et al., 2006). Therefore, it is vital to determine nickel at trace levels in wastewater and water with regard to the environment and public health (Jiang et al., 2008). Electrothermal atomic absorption spectrometry (ET-AAS), inductively coupled plasma emission spectrometry (ICP-AES), flame atomic absorption spectrometry (FAAS), and inductively coupled plasma-mass spectrometry (ICP-MS) are among the most usual analytical approaches “Zachariadis et al. (2002), Koksal et al. (2002), Cabon (2002), De Mattos et al. (2005)” for trace determination of Ni (II) ions (Ndungu et al., 2004; Mirabi et al., 2015). However, relatively high-cost apparatus and higher instrumentation complexity are required by all aforementioned approaches (except FAAS), which limit their extensive application to routine analytical work. FAAS is widely used to determine Ni (II) ions, owing to its friendly operation, low cost, good selectivity, and high sample throughput (Gonzales et al., 2009). Several difficulties exist in determining trace quantities of Ni (II) ions in wastewater and water samples by FAAS owing to inadequate tool sensitivity or matrix interferences. It is difficult to directly determine very low concentration levels of nickel in numerous environmental samples (typically ng/mL or sub–ng/mL) without preconcentration. Hence, several preconcentration methods are normally used before nickel measurement, including liquid–liquid extraction (LLE), cloud-point extraction (CPE), solidified floating organic drop microextraction (SFODME), solid-phase extraction (SPE), floatation, and ion exchange (Rezaei and Nedjate, 2003; Tuzen et al., 2006; Karami et al., 2008; Lemos et al., 2008; Shirani et al., 2009; Siadati and Mirabi, 2015; Mirabi et al., 2017a; Mirabi et al., 2018; Sadeghi et al., 2019; Bahalkeh et al., 2020; Movaghgharnezhad et al., 2020). SPE possesses some benefits over other preconcentration methods, including a higher concentration factor, ease of automation, and simple usage. The SPE method needs a powerful adsorbent. Nanomaterials can present numerous advantages over traditional SPE sorbents, like a short diffusion route and very large surface areas, leading to higher efficiency and extraction capacity (Souza and Tarley, 2009; Mirabi et al., 2017b). Abundant substances were presented and used as SPE sorbents like Diaion HP-20 (Soylak, 2002), cellulose (Gustavo et al., 2004), activated carbon (Ghaedi et al., 2007), Lewatit S 100 (Gode and Pehlivan, 2006), SDS-coated alumina (Mirabi et al., 2016), polyurethane foam (Saeed and Ahmed, 2006), Chelex 100 (Manouchehri and Bermond, 2006), microcrystalline naphthalene (Cesur and Bati, 2002), modified silica (Mirabi and Hosseini, 2012), and Diaion HP-2MG (Tuzen and Soylak, 2004). Mesoporous silica can present various advantages over traditional SPE sorbents, like very large surface areas, selectivity, and thermal stability, leading to higher efficiency and extraction capacity. In this work, the analytical potential of SBA-15 mesoporous silica was investigated, modified with 1-(2-thiazolylazo)-2-naphthol (TAN) as an adsorbent (to separate and preconcentrate trace amounts of nickel ions). This technique is explained in detail in the experimental section. It was used for measuring nickel ions in wastewater and natural water samples.

## Materials and methods

### Reagents and apparatus

Ethanol, nickel (II) nitrate, Pluronic P123, sodium hydroxide, and tetraethyl orthosilicate of analytical grade were bought from Merck. To prepare the nickel stock solution (1000.0 mg L^-1^), suitable quantities of nickel (II) nitrate hexahydrate (99.9%) were dissolved in the ultrapure water. The stock solution was used in serial dilutions with ultrapure water to make working solutions. TAN was provided as a surface modifier (ligand) by Sigma Aldrich. To adjust pH and precipitate nanocomposite, a Metrohm 744 pH Meter and centrifuge were utilized, respectively. Ni (II) ions concentration was measured via a Thermo FAAS (Model: M5) (lamp current, 15 mA; monochromator spectral band pass, 0.1 nm; burner head = 50 mm; acetylene and air-flow rates: 0.8 and 10.0 L min^-1^, respectively). The diameter of the fibers was determined using an HF2000 (Hitachi) Transmission Electron Microscope. The surface area was evaluated using the Brunauer–Emmett–Teller (BET) technique via Quanta Chrome (Chem BET 300 TPR/TPD). For elemental analysis of nanocomposites, energy-dispersive X-ray spectroscopy (EDS) was carried out using the Mira 3-XMU tool. To prove surface functionalization, elemental analysis CHNS (Costech ECS4010, Italy), Fourier-transform infrared (FT-IR) (Agilent Resolutions pro), and thermogravimetric analysis (TGA) devices (Bahr) were utilized. To produce SBA-15, Whatman #1 filter paper was utilized as a starting material.

## SBA-15 nanoparticle preparation

SBA-15 was synthesized via a known process (Yu et al., 2002) using a combination of triblock copolymer, EO20PO70EO20 (Pluronic P123, BASF) (0.02 mol), HCl (6 mol), tetraethyl orthosilicate (TEOS, 98%, Acro) (1 mol), H_2_O (166 mol), and KCl (1.5 mol). Approximately 12 g of P123 was dissolved in a combination of distilled water (375.6 g) and concentrated HCl (74.4 g) at 38°C by adding 16.5 g of KCl. Then, TEOS (31.5 g) was inserted into the solution while stirring vigorously for 8 min. For 24 h, the mixture was statically kept at the same temperature and then moved to Teflon-lined autoclaves and kept for another 24 h in an oven at 130°C. The solid was recovered by filtration and rinsed with water. Then, by refluxing with an EtOH solution, the surfactant was extracted via a Soxhlet extractor for 36 h. The gained SBA-15 was dried at 100°C overnight.

### Modification of the TAN/SBA-15

**FIGURE 1:**
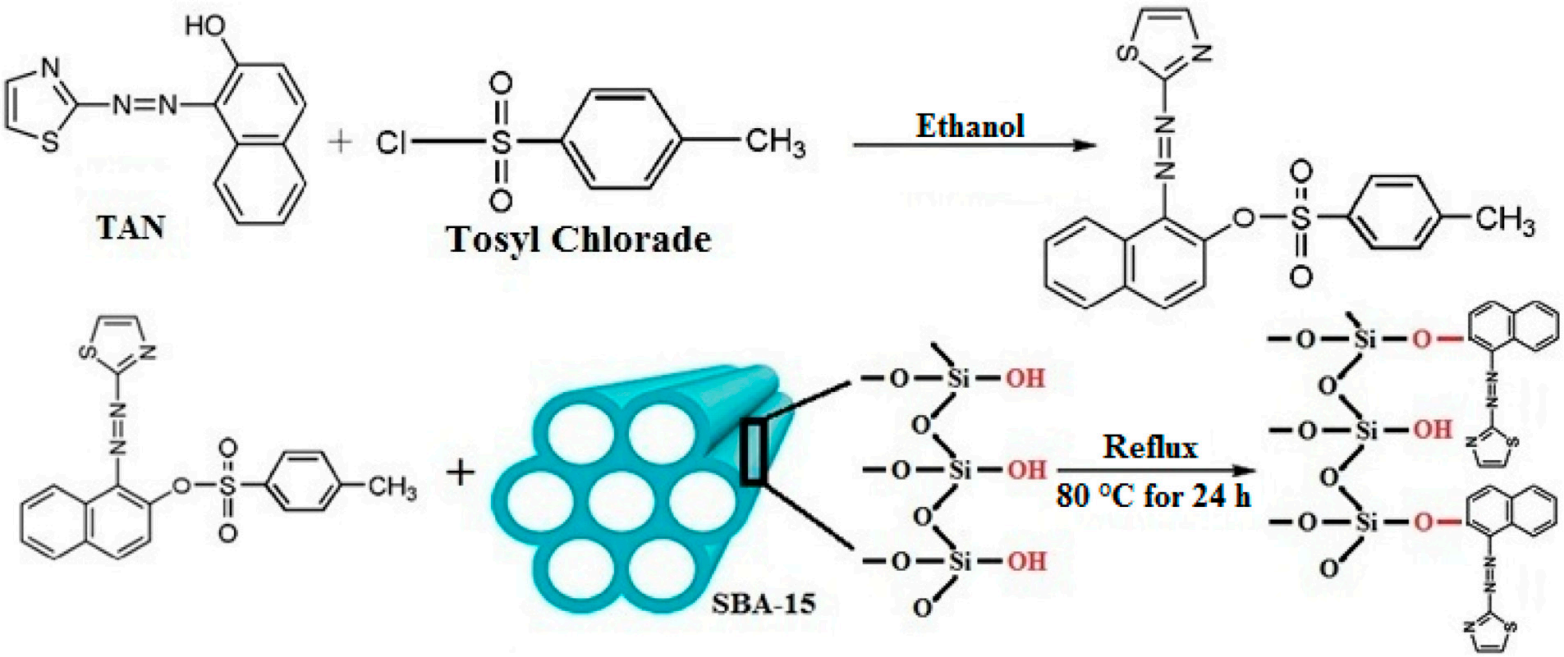
Preparation of TAN/SBA-15.

After adding 2.0 g TAN and 4.7 g tosyl chloride to ethanol (50 mL), the mixture was put in an ice bath at 5°C and mixed for 45 min. Next, the mixture was stirred for 48 h at 25°C. Stripping off the ultimate precipitated sample using a paper filter, it was rinsed with distilled water and ethanol several times and dried for 1 week at 50°C to create TAN-tosylate. A measure of 1.0 g of TAN-tosylate, 40 mL of ethanol, and 3.0 g of SBA-15 were put under reflux conditions for 24 h at 80°C. The precipitate was then gathered using a paper filter and rinsed several times. Hence, a lack of unreacted reagent was ensured on the sample surface. Until the spectrometer showed no peak for TAN-tosylate absorption, the washing procedure was continued at its λ_max_. Therefore, no unreacted TAN-tosylate was found on the sample surface. Next, the sample was dried for 1 week at 50°C. Figure 1 shows the reactions for the surface modification of SBA-15.

### Extraction mechanism

The mechanism of extraction involves the formation of a chemical bond between Ni (II) ion and SBA-15/TAN. The Ni (II) ion, on one hand, forms a coordinate covalent bond with a nitrogen atom that has a double bond, and on the other hand, the Ni (II) ion should be coordinated with a heteroatom that facilitates electron loss. Sulfur is a better heteroatom because, first, its electronegativity is lower, and second, while the valence electrons of nitrogen are in 2P orbitals, the valence electrons of sulfur are in 3P orbitals, which are farther from the nucleus and easier to lose.

### General procedure of extraction

After the addition of 0.05 g of the nanocomposite to a solution (100 mL) comprising 100 ng mL^-1^ Ni (II) ions, extraction was performed. Here, a buffer solution (2.0 mL, acetic acid/sodium acetate) with pH = 6 was added to the mixture. The complete mixture was mixed in a shaker at room temperature for 10 min for the adsorption of Ni (II) ions on the nanocomposite. Then, centrifuging the solution was performed for 5 min at 4,500 rpm for phase separation. After overflowing the upper solution, the sample was eluted by dissolving Ni (II) ions adsorbed on the precipitated sorbent with 1.0 mL HNO_3_ (0.2 mol L^-1^). After adding HNO_3_ and shaking the mixture for 5 min, the solution was re-centrifuged (for 10 min at 5,500 rpm). Finally, the Ni (II) ion concentration was measured by FAAS.

## Results and discussion

### Characterizing SBA-15 and TAN/SBA-15

It is clear that the active sites and effective surface area are provided by the synthesis of SBA-15 mesoporous silica in nanoscale to functionalize by chemical reagents. According to BET analysis for SBA-15, the specific surface area of SBA-15 is 364.2 m^2^/g, which decreases to 327.5 m^2^/g, followed by surface modification. As stated in the former section, the solid-phase extraction orients the preconcentration of Ni (II) ions through adsorption over the modified SBA-15 active sites. Hence, high surface area and porous structure are satisfactory parameters for better adsorption. The details of the BET technique for the prepared modified SBA-15 represent a more precise surface area than for the modified SBA-15 samples in the literature (Khanjari et al., 2018).

**FIGURE 2:**
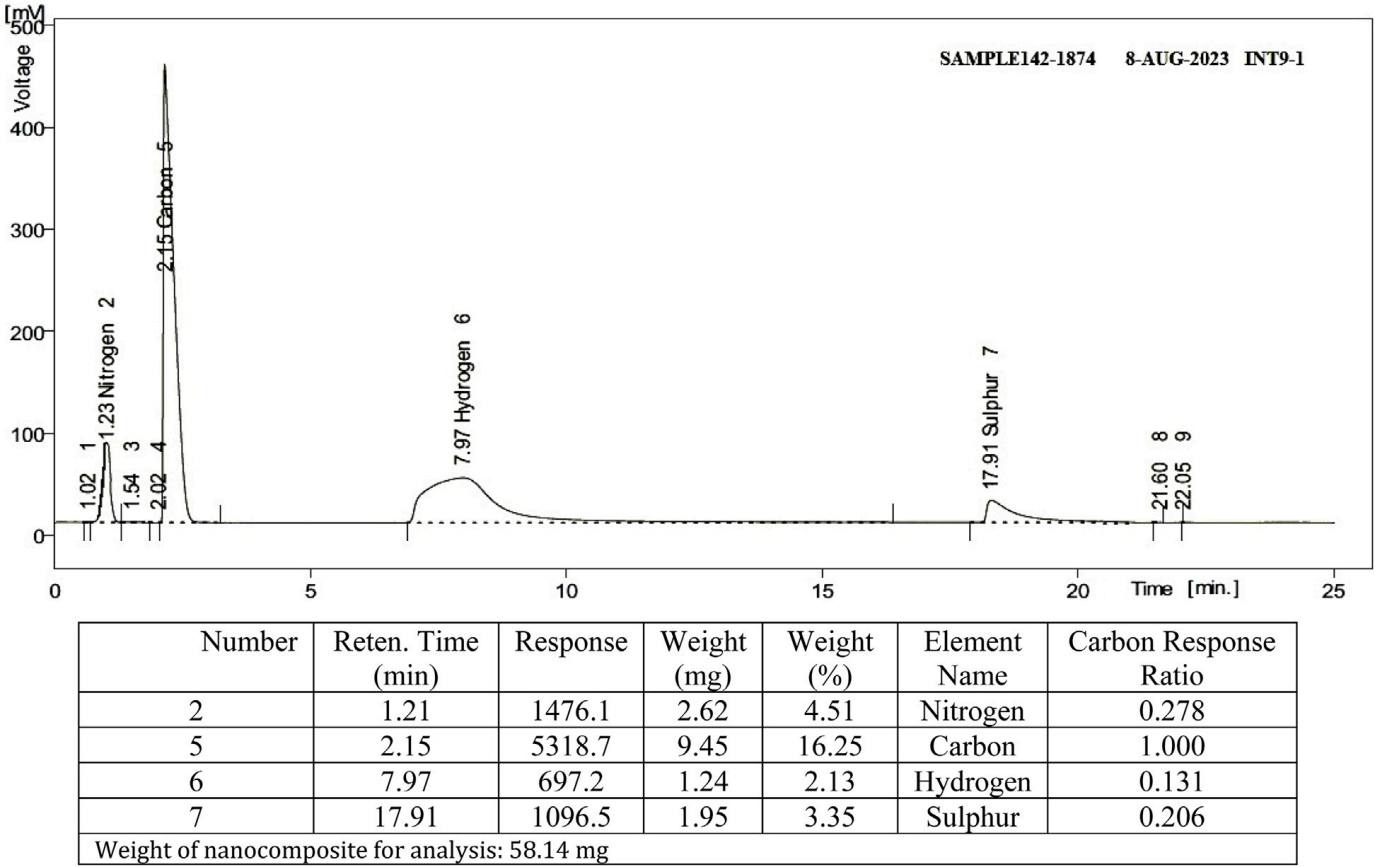
Results of CHNS elemental analysis.

Using elemental analysis CHNS, the types of elements in the nanocomposite were distinguished along with the element ratio within the sample. The existence of TAN ligand on SBA-15 was confirmed through CHNS analysis. Figure 2 shows that the nanocomposite contains the N, C, and S elements associated with TAN in the ratios mentioned. As Si, H, and O elements exist in SBA-15 along with N, C, and S in the nanocomposite, TAN is fixed over SBA-15.

**FIGURE 3:**
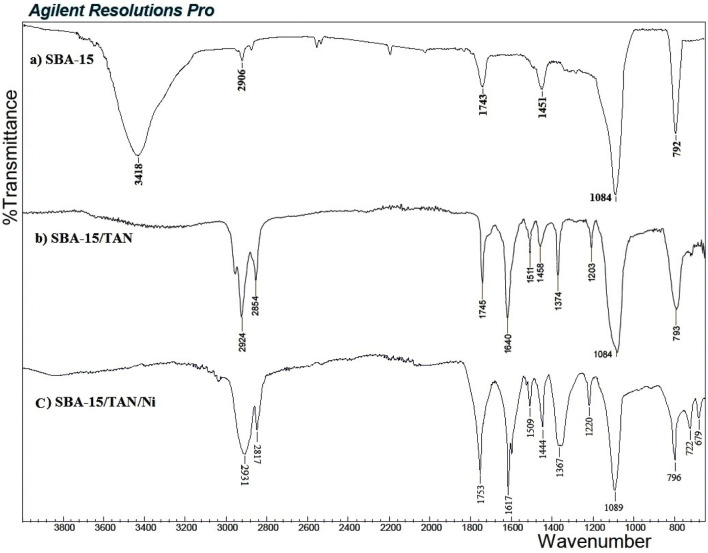
FT-IR spectrum of SBA-15 **(A)**, SBA-15/TAN nanocomposite **(B)**, and SBA-15/TAN/Ni **(C)**.

Using FT-IR, the bond formation between TAN and SBA-15 is confirmed (Figure 3). Comparing the FT-IR spectra of SBA-15 with TAN/SBA-15, the immobilization of TAN ligand on SBA-15 is significantly recognized. The broad absorption band at 3,418 cm^-1^ is associated with the OH group’s stretching vibration over the SBA-15 surface. The absorption band at 2,906 cm^-1^ is allocated to the CH bond symmetric stretching vibration. The absorption band at 1451 cm^-1^ is related to the C-H bond vibration of the CH_2_ group, while the absorption band at 1084 cm^-1^ represents the Si-O bond of SBA-15 (Figure 3A). The peaks of N=N bond at 1511 cm^-1^, C–N bond at 1203 cm^-1^, C=C bond at 1620 cm^-1^, and C–O bond at 1374 cm^-1^ can be found in the FT-IR spectra of TAN/SBA-15 (Figure 3B), leading to the TAN attachment to the SBA-15. After the extraction, absorption bands at 679 cm^-1^ and 722 cm^-1^ are related to the vibration of S-Ni and S-Ni bonds of SBA-15/TAN/Ni, respectively (Figure 3B).

**FIGURE 4:**
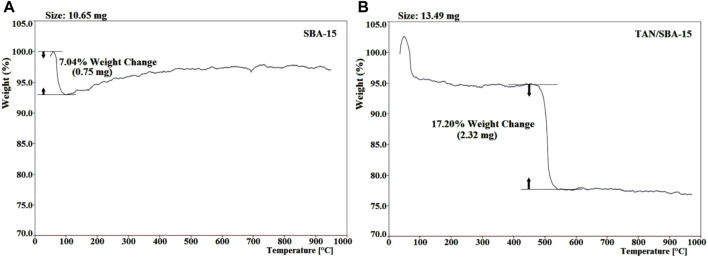
TGA of SBA-15 **(A)** and nanocomposite **(B)**.

The thermogravimetric study of TAN/SBA-15 was performed to assess the nanocomposite stability and the existence of organic groups in the material (Figures 4A, B). Approximately 10.65 mg of SBA-15 and 13.49 mg of TAN/SBA-15 were heated at 20 °C/min in an argon atmosphere. The existing water and TAN ligand are responsible for weight loss of 7.04% at approximately 95°C (Figure 4A) and weight loss (17.20%) between 480°C and 530°C (Figure 4B), respectively. A small weight and continuous loss are observed at T > 550°C, associated with the combustion of residual organic material. As observed, the TAN structure has less thermal stability than the structure of nanocomposites.

**FIGURE 5:**
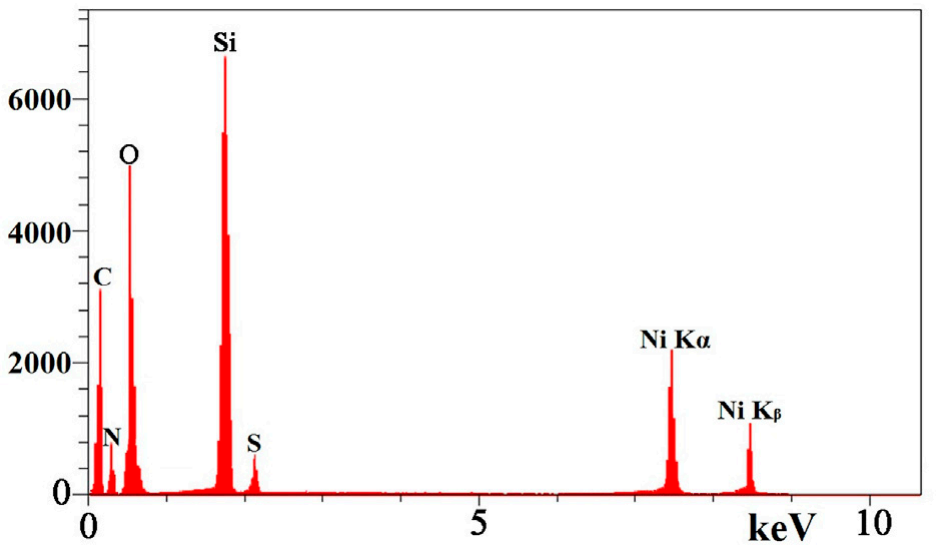
EDS spectrum of TAN/SBA-15.

These findings are in line with the results of the EDS analysis. EDS analysis of TAN/SBA-15 (Figure 5) represents the presence of C, S, and N atoms, indicating that TAN is bonded to the external surface of SBA-15 and confirming the good purity of the sample. Moreover, Ni (II) ions are demonstrated in Figure 5 chelated by TAN ligand in the nanocomposite, followed by the adsorption experiment.

**FIGURE 6:**
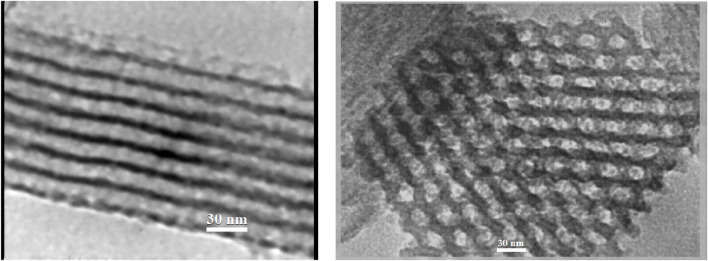
TEM image of SBA-15.

The SBA-15 meso cavity contains holes with a completely identical arrangement and a regular 6-sided 2D arrangement. A structural pattern is observed with a certain order (Figure 6), associated with the TEM images for SBA-15. The cavity size in the structure is approximately 25–30 nm. It is proved that the synthesized SBA-15 has nanoscale dimensions.

### Improvement of the extraction stage

The effects of some parameters were also studied on preconcentration and extraction. In all experimental circumstances, the concentration of Ni (II) ions was 100 ng mL^-1^.

### Effects of pH

**FIGURE 7:**
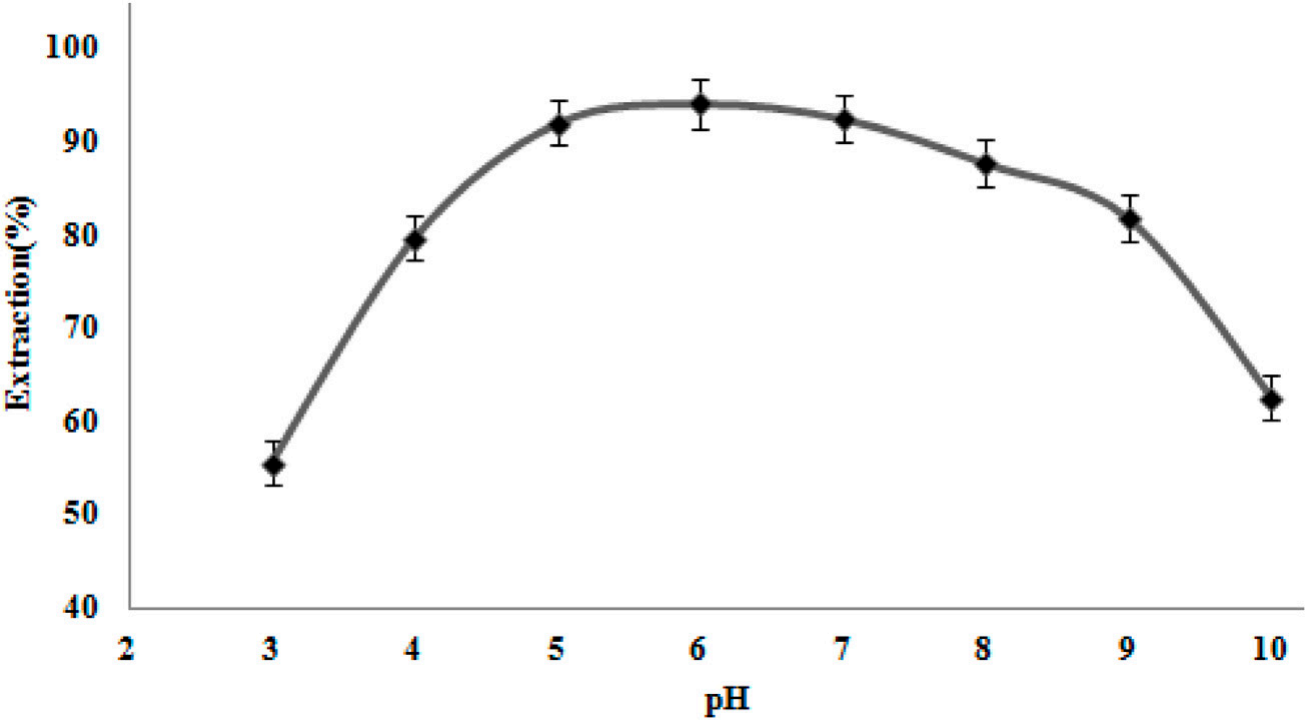
Effect of pH on Ni (II) ion extraction. Extraction circumstances: amount of nanocomposite, 50 mg; eluent type, nitric acid (0.2 M); extraction time, 10 min; recovery time, 5.0 min.

The effects of pH on Ni (II) ion extraction were studied at pH values of 3.0–10.0 while keeping other parameters constant (Figure 7). As the pH of the solution increases, the recovery of Ni (II) improves, with effective recovery obtained at a pH of 5.0–7.0. At lower pH values, the nanocomposite’s surface seems to have a positive charge caused by protonation. Hence, the electrostatic adsorbent between the Ni (II) ions and nanocomposite decreased, resulting in the maximum amount of absorption in a mild acidic solution. At higher pH values, recovery is reduced, probably owing to the nickel hydroxide formation. Thus, by adding the acetate/acetic acid buffer solution, further extraction was conducted at pH 6.0.

### Effect of the amount of nanocomposites

**FIGURE 8:**
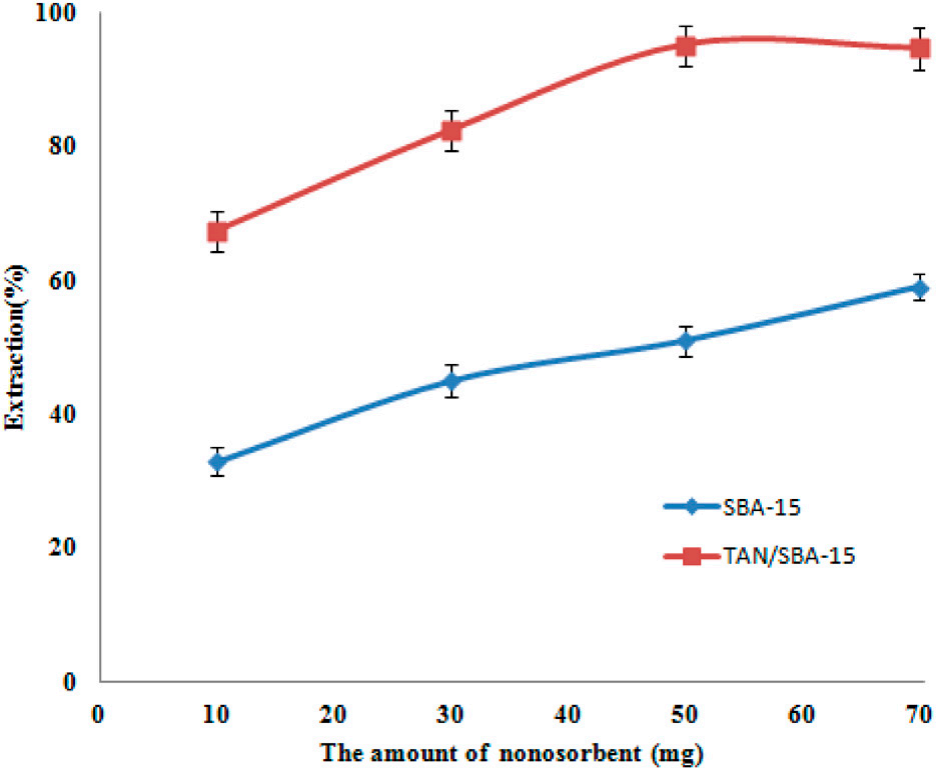
Effects of amount of nanosorbents on Ni (II) ion extraction. Extraction conditions: pH value, 6.0; eluent type, nitric acid (0.2 M); extraction time, 10 min; recovery time, 5.0 min.

The nanocomposite sorbents usually have a larger surface than usual sorbents (Mirabi et al., 2017b). Hence, a more desirable outcome could be obtained by utilizing fewer amounts of nanocomposite sorbents. The effect of several quantities of nanosorbents (SBA-15 and TAN/SBA-15) on the amount of nickel ion sorption was studied on the 10–70 mg scale. Figure 8 shows that using a higher amount than 50 mg of the modified SBA-15 nanocomposite surfaces could lead to a higher amount of nickel ion recovery. Hence, 50 mg was chosen as the optimum concentration of this sorbent. Moreover, good efficiency was not represented by purely SBA-15 when applied separately.

### Influence of extraction time

**FIGURE 9:**
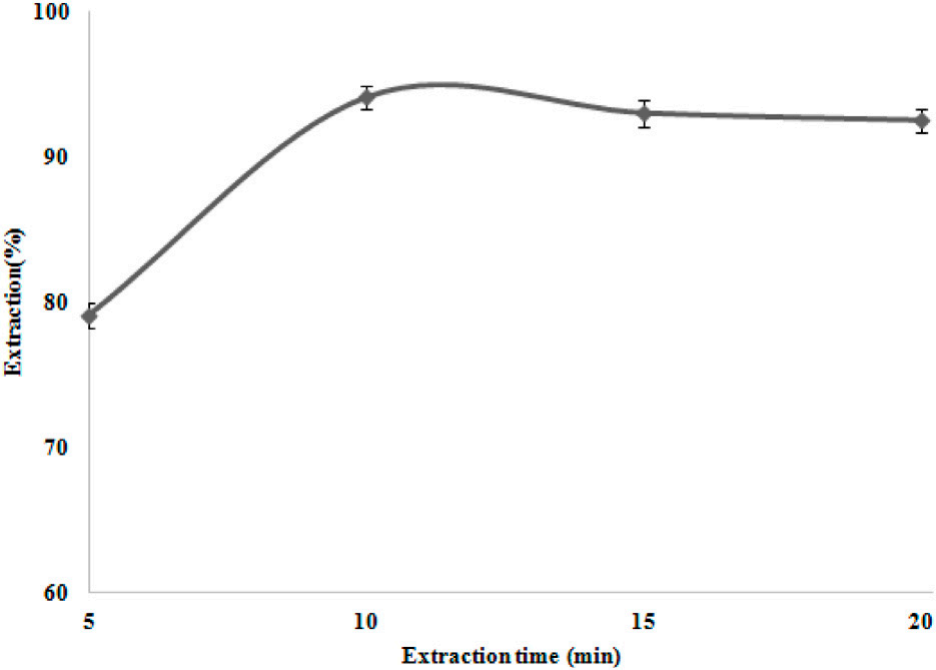
Extraction time effect on Ni (II) ion extraction. Extraction conditions: pH value, 6.0; eluent type, nitric acid (0.2 M); amount of nanocomposite, 50 mg; and recovery time, 5.0 min.

A good adsorbent achieves preconcentration in a short time. Hence, the rate of Ni (II) ion adsorption by modified nanocomposites was investigated with 0.05 g of the TAN/SBA-15 over a series of various shaking times (5–20 min). According to Figure 9, the absorbance of the Ni (II) ions showed no considerable variation after 10 min. Therefore, for further studies, the extraction time of 10 min was chosen. The ligand could create more complexes with nickel ions by increasing the time. Nevertheless, ion desorption is possible over a longer period, thus slightly reducing the extraction percentage.

### Effect of the eluting solution condition

A higher enrichment factor is obtained using an appropriate eluent. Hence, various experiments were used to select a proper solvent for the desorption of Ni (II) ions from the modified nanocomposites. The Ni (II) ions were desorbed with diverse concentrations (0.1–0.5 mol L^-1^) of 1.0 mL of various acids. The results showed (Table 1) that HNO_3_ 0.2 mol L^-1^ achieved the quantitative elution of the target analyte chelated with TAN. Therefore, we have chosen 1.0 mL of HNO_3_ (0.2 mol L^-1^) as the solvent for Ni (II) ion desorption.

**TABLE 1:** Effects of concentration and type of eluent on extraction. Extraction conditions: pH value, 6.0; extraction time, 10 min; amount of nanocomposite, 50 mg; and recovery time, 5.0 min.

Eluent	Concentration (mol L^-1^)	Extraction (%)
HCl	0.1 0.2 0.5	83.4 87.2 92.0
HNO_3_	0.1 0.2 0.5	89.3 95.3 95.4
H_2_SO_4_	0.1 0.2 0.5	79.7 73.8 75.9
CH_3_COOH	0.1 0.2 0.5	51.3 61.0 63.6

### Nanocomposite ability for reusing

Adsorbent reusability is a key factor in analytical approaches. For absorption, the adsorption capability was assessed several times. It was indicated that TAN/SBA-15 can be utilized, followed by solvent recovery in the Ni (II) ion extraction process. The procedure was replicated consecutively, with a reduction in the extraction percentage (86.6%) from the order of 3 onwards and a maximum extraction percentage (96.3%–93.6%). The extraction reduction happens owing to nanocomposite particle loss during elution, irreversible/strong interactions between Ni (II) ions and TAN ligand, and releasing the TAN ligand grafts. Hence, the adsorbent can be utilized three times successively for extracting the Ni (II) ions.

### Effect of foreign ions

Matrix interference is the main limitation to the FAAS method to determine heavy metal ions. The effects of some ions on the recovery of the analyte ions were studied at different concentrations. For Ni (II) ions, the extraction (100 mL) of solutions comprising 100 ng mL^-1^ of Ni (II) ions and different quantities of interfering ions was studied (Table 2). According to the results, there is a tolerance limit of < ± 5%. Thus, the recovery of Ni (II) ion extraction is not affected by the existence of foreign ions via TAN/SBA-15. The reason is that the ligand functions as a selective sorbent for nickel ions, and a nickel lamp is utilized as an atomic absorption tool. Hence, it can be selective largely for nickel ions.

**TABLE 2:** Effects of some foreign ions on Ni (II) ion recovery.

Interfering	Added as	Interference/Ni (II) (weight ratio)	Recovery (%)
Na^+^	NaNO_3_	500	97.4 ± 1.6
Ca^2+^	Ca(NO_3_)_2_	500	96.5 ± 1.3
Cu^2+^	CuCl_2_	100	98.1 ± 0.9
Ag^+^	AgNO_3_	50	95.9 ± 1.4
Mn^2+^	MnCl_2_	50	96.5 ± 1.5
Zn^2+^	ZnSO_4_	40	97.2 ± 1.7
Pb^2+^	PbSO_4_	30	98.1 ± 0.8
Co^2+^	Co(NO_3_)_3_	30	96.3 ± 1.3
Hg^2+^	HgCl_2_	30	95.5 ± 1.6
Fe^2+^	FeSO_4_	30	97.2 ± 1.9
Al^3+^	Al_2_(SO_4_)_3_	20	96.4 ± 1.2
Cr^3+^	Cr(NO_3_)_3_	20	95.8 ± 1.5
NO^3-^	NaNO_3_	500	98.2 ± 0.9
Cl^−^	NaCl	200	97.6 ± 1.3
SO_4_^2-^	NaSO_4_^2-^	200	97.1 ± 1.4

### Validating the technique

**FIGURE 10:**
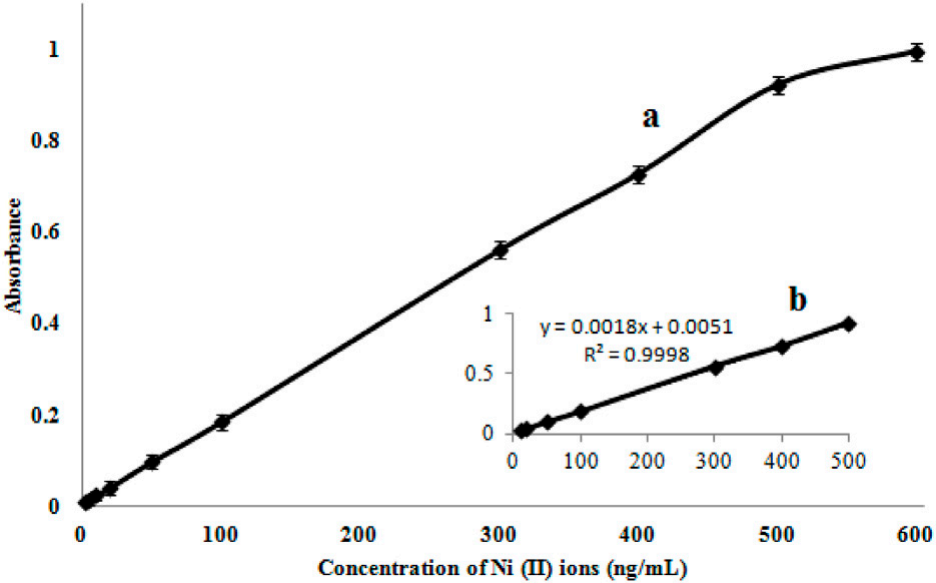
Calibration curve **(A)** and its linear range at lower concentrations **(B)**.

Using the suitability values for each technique, the efficiency is compared with other similar approaches (Table 3). The calibration curve has a linear dynamic range (LDR) of 5.0–500 ng mL^-1^ and a limit of detection (LOD) of 1.8 ng mL^-1^ (Figure 10). Moreover, to obtain a preconcentration factor (PCF) of 100, a sample volume ratio of 100 mL was considered, with a disintegration solvent volume (1 mL), and the enrichment factor (EF = 100) was derived from the concentration ratio of the Ni (II) ions, followed by the extraction to the pre-extraction concentration. The extraction percentage is approximately 100, and the values of EF and PCF are approximately equivalent in some cases, like the present work.

**TABLE 3:** Analytical properties of the presented technique.

Parameter	Analytical feature
Linear range (ng mL^-1^)	5.0–500
Regression equation	y = 0.0018 X + 0.0051
Correlation coefficient (R^2^)	0.9998
Detection limit (ng mL^-1^)	1.8
Repeatability (%)	1.0–2.9
Intermediate precision (%)	1.7–4.2
Preconcentration factor	100
Enrichment factor	100

### Using the technique

The capability of the presented technique for complex matrix intolerance was investigated using this technique for measuring Ni (II) ions in wastewater and natural water specimens. The accuracy of the technique was verified using the standard increase technique (Table 4). As observed, the method’s extraction efficiency was not highly affected by the complex matrices.

**TABLE 4:** Determination of Ni (II) ions in real specimens.

Sample	Spiked ng/mL	Found ng/mL	Recovery% (n = 3)
Tap water (from the drinking water system of Behshahr, Iran)	—	7.1	—
	100	104.0	97.1 (±1.9)
Sea water (Caspian sea, Iran)	—	75.6	—
	100	168.4	95.9 (±2.8)
River water (Telar River, Qaem Shahr, Iran)	—	94.8	—
	100	190.1	97.6 (±2.6)
Well water (Behshahr, Iran)	—	11.5	—
	100	109.8	98.5 (±3.1)
Wastewater from the Acrytab Textile Factory (Behshahr, Iran)	—	19.7	—
	100	121.1	101.2 (±2.6)
Wastewater from the Behpak Industrial Company (Behshahr, Iran)	—	23.5	—
	100	120.2	97.3 (±3.3)
Wastewater from the electrical power station (Naka, Mazandaran, Iran)	—	65.1	—
	100	158.0	95.7 (±2.8)
Wastewater from the MDF factory (Arian chemistry company, Sari, Iran)	—	83.7	—
	100	177.8	96.8 (±2.6)

## Comparison with other approaches

A comparison was made between the presented technique and the different approaches used presently to preconcentrate and measure Ni (II) ions (Table 5). Comparing the suitability of the proposed technique with other approaches indicated a lower LOD and a higher preconcentration factor for this technique.

**TABLE 5:** Comparison of the present study with former reports.

Adsorbent	Sample volume (mL)	LOD (ng mL^-1^)	PCF	Sample	Reference
mMWCNT@PADAP	20	7.1	5	Tobacco	Soylak et al. (2022)
Fe_3_O_4_@Diaion@PADAP	—	15.0	100	CRM	Soylak et al. (2023)
(Zn-Al LDH)-(PTh/DBSNa)-Fe_3_O_4_	10	1.3	40	Beef, fish meat, hen, orange plant, apple plant, and banana plant	Rajabi et al. (2020)
Fe_3_O_4_@SiO_2_@polypyrrole	750	1.2	—	Seafood mix	Abolhasani et al. (2015)
Magnetite coated with cationic surfactant sodium dodecyl sulfate	100	3.9	100	Water and herbal ervas	Mirabi et al. (2015)
TAN/SBA-15	100	1.8	100	Natural water and wastewater	This work

mMWCNT: magnetic multiwalled carbon nanotubes; PADAP: 2-(5-bromo-2-pyridylazo)-5-diethylamino-phenol; (Zn-Al LDH)-(PTh/DBSNa)-Fe_3_O_4_: Zn–Al-layered double hydroxide (Zn-Al LDH) combined with polythiophene (PTH)/sodium dodecyl benzene sulfonate (DBSNa) and Fe_3_O_4_; PCF:,preconcentration factor; LOD, limit of detection; CRM, certified reference material.

## Conclusion

SPE appears to be a powerful technique for preparing specimens. TAN/SBA-15 is utilized as a solid phase in the SPE technique. Using SBA-15 modified with a TAN ligand to extract Ni (II) ions from the solution increases the absorption capacity, thereby selectively absorbing Ni (II) ions. In the present work, the samples were analyzed using flame atomic absorption spectrometry, demonstrating that the technique is simple and possesses minimal properties. It was revealed that the modified nano-adsorbent has a higher sensitivity for measuring Ni (II) ions in lower concentrations. Moreover, it is highly effective in analyzing wastewater and natural water samples. The proposed SPE technique possesses a wide linear range, small RSD, high preconcentration factor, low LOD, and high enrichment factor.

## Data Availability

The original contributions presented in the study are included in the article/Supplementary Material; further inquiries can be directed to the corresponding author.
