# Supplementary material for: Synthesis of the modified SBA-15 mesoporous silica with TAN ligand to preconcentrate and determine trace amounts of Ni (II) ions in water and wastewater samples
Source: Front Chem. 2024 Sep 30;12:1410136. doi: 10.3389/fchem.2024.1410136 (PMC11494652; doi:10.3389/fchem.2024.1410136)
Supplement: Supplementary file 1 [file DataSheet1.docx]

**Synthesis of the modified SBA-15 mesoporous silica with TAN ligand to preconcentrate and determine trace amounts of Ni (II) ions in water and wastewater samples**

Taha Amouzad Mahdirajeh^1^, Ali Mirabi^2*^, Mohammad Habibi Juybari^1^, Ramin Zafar Mehrabian^1^ and Hamid Reza Jalilian^1^

*^1^Department of Chemistry, Islamic Azad University, Gorgan, Iran, 2Department of Chemistry, Islamic Azad University, Qaemshahr, Iran*

**Supplementary Table S1.** Effect of pH

| Error Bar | Extraction (%) | Absorption | pH |
| --- | --- | --- | --- |
| 2.5 | 55.6 | 0.104 | 3 |
| 2.3 | 79.7 | 0.149 | 4 |
| 2.4 | 92.0 | 0.172 | 5 |
| 2.7 | 94.1 | 0.176 | 6 |
| 2.6 | 92.5 | 0.173 | 7 |
| 2.5 | 87.7 | 0.164 | 8 |
| 2.6 | 81.8 | 0.153 | 9 |
| 2.4 | 62.6 | 0.117 | 10 |

**Supplementary Table S2.** Effect of the amount of nanocomposite

| Extraction (%) | Amount of  TAN/SBA-15 (mg) | Extraction (%) | Absorption | Amount of  SBA-15 (mg) |
| --- | --- | --- | --- | --- |
| 67 | 10 | 33 | 0.126 | 10 |
| 82 | 30 | 45 | 0.154 | 30 |
| 95 | 50 | 51 | 0.178 | 50 |
| 95 | 70 | 59 | 0.177 | 70 |

**Supplementary Table S3.** Influence of extraction time

| Error Bar | Extraction (%) | Absorption | Extraction Time (min) |
| --- | --- | --- | --- |
| 0.9 | 79.1 | 0.148 | 5 |
| 0.8 | 94.1 | 0.176 | 10 |
| 0.9 | 93.0 | 0.174 | 15 |
| 0.8 | 92.5 | 0.173 | 20 |

**Supplementary Table S4.** The effect of eluting solution condition

| Eluent | Concentration (mol L^-1^) | Absorption | Extraction  (%) |
| --- | --- | --- | --- |
| HCl | 0.1  0.2  0.5 | 0.156  0.163  0.172 | 83.4  87.2  92.0 |
| HNO_3_ | 0.1  0.2  0.5 | 0.167  0.178  0.179 | 89.3  95.2  95.7 |
| H_2_SO_4_ | 0.1  0.2  0.5 | 0.149  0.138  0.142 | 79.7  73.8  75.9 |
| CH_3_COOH | 0.1  0.2  0.5 | 0.096  0.114  0.119 | 51.3  61.0  63.6 |

**Supplementary Table S5.** The nanocomposite ability for reusing

| Extraction (%) | Absorption | Order of reuse |
| --- | --- | --- |
| 96.3 | 0.180 | First time |
| 95.2 | 0.178 | Secon time |
| 93.6 | 0.175 | Third time |
| 86.6 | 0.162 | Fourth time |
| 72.2 | 0.135 | Fifth time |

**Supplementary Table S6.** Effect of foreign ions

| Interfering | Added as | Interference/Ni (II)  (weight ratio) | Absorption | Recovery  (%) |
| --- | --- | --- | --- | --- |
| Na^+^ | NaNO_3_ | 500 | 0.182 | 97.4 ± 1.6 |
| Ca^2+^ | Ca(NO_3_)_2_ | 500 | 0.180 | 96.5 ± 1.3 |
| Cu^2+^ | CuCl_2_ | 100 | 0.183 | 98.1 ± 0.9 |
| Ag^+^ | AgNO_3_ | 50 | 0.179 | 95.9 ± 1.4 |
| Mn^2+^ | MnCl_2_ | 50 | 0.180 | 96.5 ± 1.5 |
| Zn^2+^ | ZnSO_4_ | 40 | 0.181 | 97.2 ± 1.7 |
| Pb^2+^ | PbSO_4_ | 30 | 0.183 | 98.1 ± 0.8 |
| Co^2+^ | Co(NO_3_)_3_ | 30 | 0.180 | 96.3 ± 1.3 |
| Hg^2+^ | HgCl_2_ | 30 | 0.179 | 95.5 ± 1.6 |
| Fe^2+^ | FeSO_4_ | 30 | 0.181 | 97.2 ± 1.9 |
| Al^3+^ | Al_2_(SO_4_)_3_ | 20 | 0.180 | 96.4 ± 1.2 |
| Cr^3+^ | Cr(NO_3_)_3_ | 20 | 0.179 | 95.8 ± 1.5 |
| NO^3-^ | NaNO_3_ | 500 | 0.184 | 98.2 ± 0.9 |
| Cl^-^ | NaCl | 200 | 0.182 | 97.6 ± 1.3 |
| SO_4_^2-^ | NaSO_4_^2-^ | 200 | 0.181 | 97.1 ± 1.4 |

**Supplementary Table S7.** Calibration curve

| Error Bar | Absorption | Concentration of Ni (II) ions (ng mL^-1^) |
| --- | --- | --- |
| 0.009 | 0.013 | 3 |
| 0.01 | 0.017 | 5 |
| 0.01 | 0.023 | 10 |
| 0.014 | 0.041 | 20 |
| 0.015 | 0.098 | 50 |
| 0.019 | 0.185 | 100 |
| 0.019 | 0.561 | 300 |
| 0.020 | 0.728 | 400 |
| 0.018 | 0.923 | 500 |
| 0.018 | 0.996 | 600 |

**Supplementary Table S8.** The determination of Ni (II) ions in real specimens

| Sample | Spiked  ng/mL | Absorption | Found  ng/mL | Recovery%  (n=3) |
| --- | --- | --- | --- | --- |
| Tap water (from the drinking water system of Behshar, Iran) | - | 0.018 | 7.1 | - |
|  | 100 | 0.192 | 104.0 | 97.1 (±1.9) |
| Sea water  (Caspian sea, Iran) | - | 0.141 | 75.6 | - |
|  | 100 | 0.308 | 168.4 | 95. 9 (±2.8) |
| River water  (Telar River, Qaemshahr, Iran) | - | 0.176 | 94.8 | - |
|  | 100 | 0.347 | 190.1 | 97.6 (±2.6) |
| Well water  (Behshar, Iran) | - | 0.026 | 11.5 | - |
|  | 100 | 0.203 | 109.8 | 98.5 (±3.1) |
| Wastewater of Acrytab textile factory (behshar, Iran) | - | 0.041 | 19.7 | - |
|  | 100 | 0.223 | 121.1 | 101.2 (±2.6) |
| Wastewater of Behpak Food industrial company (behshar, Iran) | - | 0.047 | 23.5 | - |
|  | 100 | 0.222 | 120.2 | 97. 3 (±3.3) |
| Wastewater of Electrical power station (Naka, Mazandaran, Iran) | - | 0.122 | 65.1 | - |
|  | 100 | 0.289 | 158.0 | 95.7 (±2.8) |
| Wastewater of MDF factory  (Arian chemistry company,  Sari, Iran) | - | 0.156 | 83.7 | - |
|  | 100 | 0.325 | 177.8 | 96.8 (±2.6) |
